# Learning curve in intestinal ultrasound: advancing from basic skills to advanced competencies–insights from the IUS IG-IBD Master program

**DOI:** 10.1093/ecco-jcc/jjaf223

**Published:** 2025-12-23

**Authors:** Cristina Bezzio, Luisa Bertin, Simone Saibeni, Davide Giuseppe Ribaldone, Federica Furfaro, Giovanni Maconi, Fulvia Terracciano, Elena Mazzotta, Emma Calabrese, Fabiana Castiglione, Ambrogio Orlando, Giuseppe Privitera, Sara Massironi, Francesca Zorzi, Lorena Pirola, Silvio Danese, Antonio Rispo, Flavio Caprioli, Mirella Fraquelli, Demis Pitoni, Arianna Dal Buono, Anna Testa, Massimo Claudio Fantini, Alessandro Armuzzi, Mariangela Allocca, Collaborators, Nicola Imperatore, Marta Vernero, Simona Ricciolino, Manuela Marzo, Alessia Guarino, Valentino Calvez, Alessia Todeschini, Elena Bartolini, Emanuele Orlando, Caterina Zoratti, Gaia Riguccio

**Affiliations:** IBD Center, IRCCS Humanitas Research Hospital, Rozzano, Milan, Italy; Department of Biomedical Sciences, Humanitas University, Pieve Emanuele, Milan, Italy; Department of Gastroenterology, University of Padova, Padova, Italy; Gastroenterology Unit, Rho Hospital, ASST Rhodense, Rho, Milan, Italy; Department of Medical Sciences, University of Turin, Turin, Italy; Gastroenterology and Endoscopy Unit, IRCCS Ospedale San Raffaele and Vita-Salute San Raffaele University, Milan, Italy; Department of Biomedical and Clinical Sciences, Gastroenterology Unit, ASST Fatebenefratelli-Sacco, University of Milan, Milan, Italy; Division of Gastroenterology, Fondazione IRCCS Casa Sollievo della Sofferenza, San Giovanni Rotondo, Italy; IBD Unit, IRCCS Azienda Ospedaliero-Universitaria di Bologna, Bologna, Italy; Department of Systems Medicine, Gastrointestinal Unit, University of Rome Tor Vergata, Rome, Italy; Department of Clinical Medicine and Surgery, Gastroenterology Unit, University Federico II of Naples, Naples, Italy; Department of Medicine, Inflammatory Bowel Disease Unit, A.O.O.R. “Villa Sofia-Cervello” Hospital, Palermo, Italy; IBD Center, IRCCS Humanitas Research Hospital, Rozzano, Milan, Italy; Department of Biomedical Sciences, Humanitas University, Pieve Emanuele, Milan, Italy; Gastroenterology and Endoscopy Unit, IRCCS Ospedale San Raffaele and Vita-Salute San Raffaele University, Milan, Italy; Department of Systems Medicine, Gastrointestinal Unit, University of Rome Tor Vergata, Rome, Italy; Gastroenterology, Fondazione IRCCS San Gerardo dei Tintori, Monza, Italy; Gastroenterology and Endoscopy Unit, IRCCS Ospedale San Raffaele and Vita-Salute San Raffaele University, Milan, Italy; Department of Clinical Medicine and Surgery, Gastroenterology Unit, University Federico II of Naples, Naples, Italy; Department of Pathophysiology and Transplantation, University of Milan, Milan, Italy; Gastroenterology and Endoscopy Unit, Fondazione IRCCS Ca’ Granda Ospedale Maggiore Policlinico, Milan, Italy; Department of Pathophysiology and Transplantation, University of Milan, Milan, Italy; Department of Medical Sciences, University of Turin, Turin, Italy; IBD Center, IRCCS Humanitas Research Hospital, Rozzano, Milan, Italy; Department of Biomedical Sciences, Humanitas University, Pieve Emanuele, Milan, Italy; Gastroenterology and Endoscopy Unit, IRCCS Ospedale San Raffaele and Vita-Salute San Raffaele University, Milan, Italy; Department of Medical Science and Public Health, University of Cagliari, Azienda Ospedaliero-Universitaria di Cagliari, Cagliari, Italy; IBD Center, IRCCS Humanitas Research Hospital, Rozzano, Milan, Italy; Department of Biomedical Sciences, Humanitas University, Pieve Emanuele, Milan, Italy; Gastroenterology and Endoscopy Unit, IRCCS Ospedale San Raffaele and Vita-Salute San Raffaele University, Milan, Italy

**Keywords:** inflammatory bowel disease, intestinal ultrasound, learning curve

## Abstract

**Background:**

Intestinal ultrasound (IUS) is increasingly valuable in inflammatory bowel disease (IBD) management.

**Objective:**

This study aimed to determine the learning curve for basic and advanced IUS parameters and establish the minimum number of examinations required for diagnostic proficiency.

**Design:**

We conducted a prospective, multicenter study across eight Italian tertiary IBD centers. Eight gastroenterology trainees with extensive abdominal ultrasound experience but limited IUS exposure completed standardized training comprising theoretical education, 30 supervised examinations, and 99 independent assessments. Expert sonographers independently and blindly reassessed all independent examinations using identical protocols. Interobserver agreement was quantified using Cohen’s kappa coefficients across 12 predefined categories, stratified into basic (bowel wall thickness, vascularity, stratification) and advanced (fistulas, collections, strictures) findings.

**Results:**

Following initial training, trainees demonstrated substantial baseline competency. Basic parameters achieved consistently high performance throughout the study period (from *κ* = 0.792 to *κ* = 0.842), while advanced findings showed more pronounced learning curves, improving from *κ* = 0.728 to *κ* = 0.854. Small bowel dilation exhibited the steepest learning trajectory (*κ* = 0.674 to *κ* = 0.921, 36.6% improvement, *P* = .204). Sustained primary competence (*κ* ≥ 0.8) was achieved by 37.5-62.5% of trainees for basic parameters within 99 examinations, with bowel wall stratification proving most challenging (37.5% success rate).

**Conclusion:**

This study establishes the first comprehensive, parameter-specific learning thresholds for IUS competency in IBD. Our findings demonstrate that structured training enables basic IUS proficiency within 69-112 examinations for experienced ultrasonographers, while advanced skills require extended practice. These data represent an important step toward defining evidence-based benchmarks for IUS training, supporting the development of standardized international curricula and safe clinical implementation.

## Graphical abstract

**Graphical Abstract:**
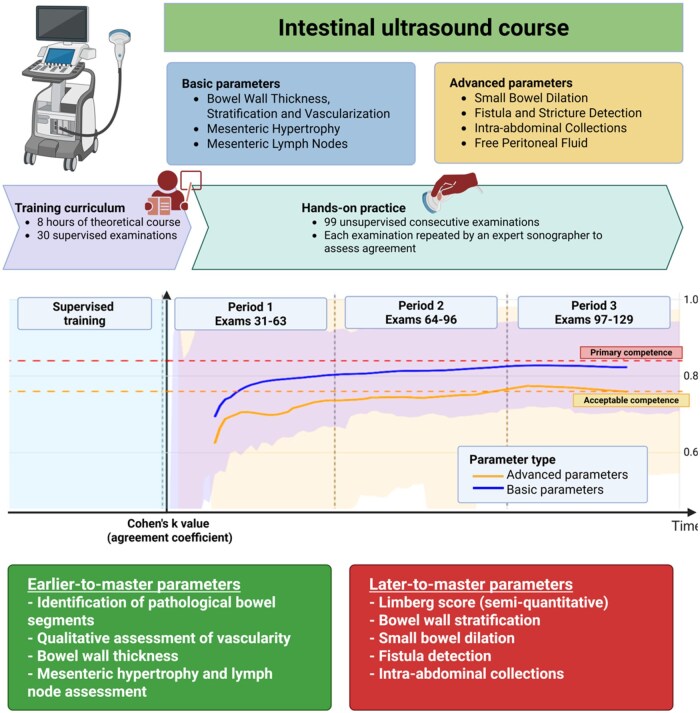

## 1. Introduction

Inflammatory bowel diseases (IBD) are chronic inflammatory conditions that require precise and frequent monitoring to optimize therapeutic strategies and improve patient outcomes.^1^^,^^2^ While endoscopy remains the gold standard for mucosal assessment, its invasiveness, cost, and inability to evaluate transmural inflammation have driven the search for complementary diagnostic modalities.^3^^,^^4^ In this context, intestinal ultrasound (IUS) has emerged as a particularly valuable non-invasive tool in the comprehensive management of IBD patients.^5^ The increasing prominence of this method is driven by several key advantages: it is a non-invasive, radiation-free, cost-effective, and widely accessible imaging modality that is well-tolerated by patients.^6-8^ Furthermore, IUS allows for real-time assessment of both intestinal and extra-intestinal structures and serves as a valuable point-of-care tool, enabling immediate clinical decision-making and disease monitoring.^9^^,^^10^

While operator dependency represents a challenge in IUS, it should be acknowledged that this characteristic is not unique to IUS, but common to all procedural diagnostic techniques. Recent evidence has demonstrated that, after appropriate training, differences between less experienced and expert operators become less pronounced, underscoring the importance of structured curricula rather than viewing operator dependence as a fundamental limitation.^11^ Moreover, studies have begun to delineate parameter-specific learning trajectories. Madsen et al. recently reported differential learning curves between bowel wall thickness (BWT) and echostratification assessment, showing that while BWT accuracy improved markedly after approximately 80 scans, echostratification remained challenging even after extensive training.^12^

Despite these advantages, the clinical utility of IUS is limited by the need for standardized training to ensure accurate and reproducible assessments. An international Delphi consensus published in 2022^13^^,^^14^ defined the core elements of IUS competency, including knowledge, image acquisition, and image interpretation, which together constitute comprehensive competence. The International Bowel Ultrasound group (IBUS) has developed standardized training curricula to address these limitations. However, evidence defining specific learning curves remains limited. Previous studies have not clearly delineated examination requirements for both basic and advanced IUS features.^15-18^

In this context, the present study focuses on the concept of competency and its measurable components, aiming to characterize parameter-specific learning curves in IUS. Specifically, our investigation aims to (1) determine the learning curve for basic versus advanced IUS parameters in IBD assessment and (2) establish the minimum number of examinations required to achieve diagnostic agreement with expert operators. Given that our participants had prior ultrasound experience, the present results should be interpreted as reflecting the learning curve of a population with existing sonographic skills, rather than novice operators.

## 2. Methods

### 2.1. Study population and design

We conducted a prospective, multicenter observational study from June 2023 to October 2024 across eight Italian tertiary IBD referral centers with expertise in IUS participating in the Italian Group for the Study of Inflammatory Bowel Disease (IG-IBD) Master program. Eight gastroenterology trainees were enrolled. All trainees had substantial experience in general abdominal ultrasound (>200 examinations), including hepatobiliary, renal, and basic abdominal assessments, but had limited specific experience with IUS (<20 examinations focused on bowel evaluation). Their prior training included FAST and POCUS protocols for emergency assessments.

Eight expert sonographers participated in the study, one at each participating center. Each trainee was paired with a single expert throughout the study period to ensure consistency in evaluation standards and each trainee–expert pair remained fixed throughout the study to ensure consistency in evaluation and feedback.

All experts had performed >2000 IUS examinations and participated in a pre-study calibration session to standardize assessment criteria. The trainees performed IUS on consecutive patients referred to the Gastroenterology Department for ultrasound examination as prescribed by their physicians.

We implemented a standardized three-phase competency-based training curriculum:

1. **Theoretical training**: an intensive 8-h module covering principles of IUS. This included didactic sessions on bowel wall anatomy, technical principles of curvilinear and linear probes, vascular Doppler parameters, and case-based image interpretation, supplemented by video-demonstrations and interactive discussion of common pitfalls.
2. **Supervised practical training**: 1 week of hands-on training under direct supervision of expert sonographers (each with >1000 IUS examinations), during which each trainee performed 30 supervised scans.
3. **Independent assessment phase:** following each examination, an expert sonographer at the same center independently reassessed all findings, blinded to the trainee’s recorded results. Although both trainee and expert performed real-time examinations, the expert did not have access to the trainee’s report, which was entered into a separate database. The expert performed their evaluation using the same equipment and standardized protocols.
4. **Initial training:** referred to the combined preparatory phase consisting of the theoretical module (8 h) and supervised practical training (30 examinations) completed before the independent assessment phase.

### 2.2. Ultrasound examination and parameters

Examinations were performed using a combination of standard low-frequency curvilinear transducers (3.5–5 MHz) and high-frequency linear transducers (7–15 MHz). Patients were examined in a supine position following European Federation of Societies for Ultrasound in Medicine and Biology (EFSUMB) guidelines.^18^ Scheduled patients were advised to fast for 6 h, and the small and large bowels were assessed under gentle probe compression.

Standardized protocols followed EFSUMB guidelines,^18^ and IBUS consensus recommendations, including: systematic assessment in a predetermined sequence (terminal ileum, cecum, ascending, transverse, descending, sigmoid colon), mandatory acquisition of both transverse and longitudinal views with minimum 10-s cine loops for each pathological segment, standardized Doppler settings (pulse repetition frequency [PRF] 800–1500 Hz, wall filter 40–60 Hz), and complete mesenteric assessment.

Although examinations were performed in real-time, standardized data collection forms ensured systematic documentation of all evaluated parameters.

For the purpose of this study, IUS *parameters* were defined as measurable sonographic features reflecting disease activity (eg, BWT, vascularity, stratification),^19^ whereas *findings* referred to qualitative abnormalities or complications (eg, abscess, fistula, free fluid).^15^

Ultrasonographic parameters were assessed and classified as either basic or advanced, incorporating the key parameters outlined by expert consensus as basic features,^19^ as well as applying evidence from our previous study.^15^

### 2.3. Basic ultrasonographic parameters

1. **Bowel wall thickness (BWT)**: measured perpendicular to the bowel wall from the hyperechoic serosa to the hyperechoic mucosal interface, with pathological threshold established at >3 mm for small and large bowel.^20^
2. **Bowel wall stratification (BWS)**: assessed as preserved (five layers clearly visualized), partially preserved or lost.^20^
3. **Vascularization qualitative assessment**: using color Doppler with standardized parameters (PRF 800-1500 Hz, wall filter 40-60 Hz; velocity scale: −7 to +7 cm/s) and classified as present or absent.^21^
4. **Vascularization semi-quantitative assessment, using the Limberg score**: the Limberg score is a semi-quantitative assessment of bowel wall vascularity:^22^ values range from 0 to 4, with higher scores indicating more marked hypervascularity.
5. **Mesenteric hypertrophy**: defined as hyperechoic thickening of perienteric fat with increased echogenicity compared to normal mesenteric fat.^20^
6. **Mesenteric lymph nodes**: pathological lymph nodes identified as oval or round hypoechoic structures >5 mm in short-axis diameter in the mesentery adjacent to affected bowel segments.^23^^,^^24^

### 2.4. Advanced ultrasonographic variables

1. **Small bowel dilation:** identification of a dilation of >25 mm in a small bowel loop.^21^
2. **Fistula detection:** detection and characterization of fistulous tracts (hypoechoic linear structures traversing from affected bowel to adjacent structures or other bowel loops), assessed with high-frequency probes.^21^^,^^25^
3. **Intra-abdominal collections:** detection of abscesses (well-defined anechoic or hypoechoic fluid collections with irregular walls, containing fluid and gaseous artifacts; posterior enhancement; sometimes within hypertrophic mesentery, and peripheral hyperemia on Doppler), and/or phlegmons (ill-defined inflammatory masses with heterogeneous echogenicity, increased vascularity, and absence of discrete fluid collection, often adjacent to affected tissues).^21^^,^^25^
4. **Free peritoneal fluid:** evaluated in all four quadrants, with particular attention to small amounts of physiological fluid often present in the pouch of Douglas (rectouterine pouch). Unlike the large-volume pathological collections assessed in FAST examinations, this parameter included detection of minimal fluid quantities that may represent normal variation or early inflammatory changes.^21^

Competency assessment was based on concordance between complete real-time ultrasound examinations performed independently by trainees and experts, rather than evaluation of static images or written reports. Both operators performed full dynamic scans, including real-time probe manipulation, systematic bowel assessment, and Doppler evaluation, with findings documented in standardized data collection forms. This study focused on interpretative competence; image acquisition technique and machine setting optimization were not formally assessed and will be addressed in future studies.

### 2.5. Statistical analysis

Baseline characteristics and pathological findings were summarized using appropriate descriptive statistics. Continuous variables are presented as mean ± standard deviation (SD) and were assessed for normality using the Shapiro–Wilk test. Between-period comparisons for continuous variables were performed using one-way ANOVA for normally distributed data or Kruskal–Wallis tests for non-parametric data. Categorical variables are presented as frequencies and percentages, with between-period comparisons conducted using chi-square tests or Fisher’s exact tests when expected cell counts were <5.

Inter-rater agreement between trainees and expert operators was assessed using Cohen’s kappa coefficient (*κ*) for all predefined ultrasound parameters. Inter-observer agreement was calculated between each trainee’s findings and the expert’s assessment, with the expert evaluation serving as the reference standard (“ground truth”). Both examinations were performed in real-time on the same day, with the expert performing their independent assessment immediately following the trainee’s examination. The expert was blinded to the trainee’s recorded results. The training protocol comprised 129 examinations per trainee: a supervised phase (examinations 1-30) under direct expert guidance, followed by an independent assessment phase (examinations 31-129) for skill evaluation. For comparative analysis of learning progression, the 99 independent examinations were stratified into three consecutive 33-examination periods. Learning curve significance was evaluated using Cochran–Armitage trend tests for individual parameters and Friedman tests for group-level comparisons. To address multiple testing, *P*-values were adjusted using the false discovery rate (FDR) method according to the Benjamini–Hochberg procedure. Simultaneously, we calculated progressive kappa coefficients from examination 31 through each subsequent examination to provide real-time competency tracking and identify precise competency achievement points. Recognizing the low prevalence of some findings, we calculated prevalence-adjusted bias-adjusted kappa values using the formula PABAK = 2 × observed agreement − 1. Parameters demonstrating |*κ* − PABAK| > 0.10 were considered to have clinically meaningful prevalence effects requiring adjusted interpretation.

Learning trajectory characterization employed both linear and logarithmic modeling approaches to capture different aspects of skill acquisition. Variables were categorized by learning difficulty based on their initial *κ* value and subsequent improvement: “Initially difficult, substantial improvement”: initial *κ* < 0.6 with absolute improvement >0.2; “Persistently difficult”: initial *κ* < 0.6 with absolute improvement ≤0.2; and “Moderate difficulty, good improvement”: initial *κ* between 0.6 and 0.8 with absolute improvement >0.1. We characterized variables as demonstrating high performance or proficiency when they achieved baseline Cohen’s kappa values exceeding 0.8 immediately after this initial training phase. Sustained primary competence was defined as the achievement of *κ* ≥ 0.80 at any point during the independent assessment phase, maintained throughout all subsequent examinations, in agreement with the competency framework proposed by the 2022 IUS Delphi consensus.^13^

Given the clustered nature of the data, mixed-effects logistic regression models were employed. For each variable, we fitted: (1) a null model with random intercepts for trainees to calculate intraclass correlation coefficients (ICCs), and (2) a period model including fixed effects for examination periods and random intercepts for trainees to assess learning curves. The ICC was calculated as τ^2^/(τ^2^ + π^2^/3), where τ^2^ represents between-trainee variance and π^2^/3 represents within-trainee variance for logistic regression models. Learning curve significance was evaluated using likelihood ratio tests comparing null and period models. The learning curve itself represented the trajectory of agreement improvement across consecutive examination periods, which we quantified through systematic changes in kappa coefficients over time. All models used the “bobyqa” optimizer with increased iteration limits (maxfun = 20 000) to ensure convergence.

We implemented a dual complementary approach to assess competency in IUS. Cumulative Sum (CUSUM) analysis with sequential probability ratio testing, using control boundaries, established acceptable and unacceptable failure rates of 5% and 20%,respectively, with Type I and Type II error rates of 5% and 10%. Competency achievement required crossing the lower boundary with maintained performance thereafter. Due to the risk of inflating CUSUM estimates in the case of low prevalence (<10%), we did not perform it in such cases. The second method was based on predefined competence benchmarks defined as primary competence (*κ* ≥ 0.80) and acceptable competence (*κ* ≥ 0.70). Competency achievement required reaching the threshold value and maintaining it throughout all remaining examinations in the assessment period.

Mixed-effects modeling was performed using hierarchical logistic regression to account for clustering within trainees. All analyses were conducted using R v.4.4.3 (R Foundation for Statistical Computing, Vienna, Austria). Statistical significance was set at *P* < .05 for all analyses. Sustained primary competence was defined as the achievement of *κ* ≥ 0.80 at any point during the independent assessment phase, maintained throughout all subsequent examinations, in agreement with the competency framework proposed by the 2022 IUS Delphi consensus.^13^

### 2.6. Sample size and power considerations

We employed an intensive repeated-measures design with eight trainees each contributing 99 consecutive examinations, yielding 792 independent assessments. The study design prioritized feasibility and comprehensive skill assessment over statistical power for detecting small effect sizes. The sample represents the complete target population for structured IUS training programs, ensuring external validity for similar educational contexts and was limited by practical training program constraints. The sample provided adequate power to detect large effect sizes in group-level analyses, though individual parameter comparisons were constrained by the small number of trainees.

### 2.7. Ethics statement

The study was conducted in accordance with the ethical standards of the *Declaration of Helsinki*. This study was conducted within the framework of a postgraduate training program (Master’s degree), which includes hands-on ultrasound practice by trainees on consenting patients as part of routine clinical care. Only anonymized, non-identifiable ultrasound parameters were recorded and analyzed. No patient-identifiable data were collected, and the findings obtained by trainees were not used for clinical decision-making.

## 3. Results

Eight trainees with prior abdominal ultrasound experience completed structured training and independently performed 99 IUS examinations each, totaling 792 assessments across three periods. Most examinations (73.3%) were for IBD monitoring, with 26.7% for suspected IBD. Within this group, the primary indications included abdominal pain (*n* = 107, 13.5%) and chronic diarrhea (*n* = 49, 6.2%), both of which represent common early clinical manifestations prompting IBD evaluation. The remaining cases (7.0%, *n* = 55) included other unspecified conditions.

### 3.1. Prevalence of pathological findings

To ensure that observed improvements in diagnostic agreement reflected genuine learning progression rather than variations in case difficulty, we assessed the consistency of patient characteristics and pathological findings across the three training periods. Patient demographics and clinical parameters remained stable throughout the study, as illustrated in Table 1. In particular, mean body mass index showed no significant variation across periods.

**Table 1.** Baseline characteristics and distribution of pathological findings across three consecutive unsupervised training periods.

Parameter	Period 1	Period 2	Period 3	*P*-value
	(*n* = 264)	(*n* = 264)	(*n* = 264)	
**Anthropometric data**				
** BMI, mean ± SD**	23.47 ± 3.59	23.16 ± 3.76	22.86 ± 3.71	.159
**Bowel wall thickness**				
** Wall thickness (mm), mean ± SD**	4.47 ± 2.22	4.45 ± 1.92	4.37 ± 1.97	.871
** Bowel wall thickening, *n* (%)**	161 (61.0)	152 (57.6)	160 (60.6)	.731
**Bowel wall stratification pattern**				
** Normal echostructure, *n* (%)**	224 (84.8)	223 (84.5)	220 (83.3)	.888
** Altered echostructure, *n* (%)**	40 (15.2)	41 (15.5)	44 (16.7)	
**Vascularization**				
** Normal vascularization, *n* (%)**	171 (64.8)	161 (61.0)	168 (63.6)	.696
** Increased vascularization, *n* (%)**	93 (35.2)	103 (39.0)	96 (36.4)	
**Limberg score**				
** Score 0, *n* (%)**	146 (55.3)	147 (55.7)	142 (53.8)	.594
** Score 1, *n* (%)**	36 (13.6)	24 (9.1)	42 (15.9)	
** Score 2, *n* (%)**	45 (17.0)	51 (19.3)	44 (16.7)	
** Score 3, *n* (%)**	22 (8.3)	27 (10.2)	26 (9.8)	
** Score 4, *n* (%)**	15 (5.7)	15 (5.7)	10 (3.8)	
**Extent of inflammation**				
** Number of involved segments, mean ± SD**	0.83 ± 0.87	1.03 ± 1.35	0.89 ± 1.13	.918
** Length of involved segments (cm), mean ± SD**	7.03 ± 10.51	7.08 ± 10.64	6.01 ± 8.96	.623
**Mesenteric features**				
** Mesenteric hypertrophy, *n* (%)**	71 (26.9)	83 (31.4)	72 (27.3)	.446
** Pathological lymph nodes, *n* (%)**	55 (20.8)	53 (20.1)	51 (19.3)	.924
**Complications**				
** Free fluid, *n* (%)**	22 (8.3)	29 (11.0)	21 (8.0)	.485
** Pathological dilation, *n* (%)**	16 (6.1)	15 (5.7)	8 (3.0)	.279
** Fistulas, *n* (%)**	8 (3.0)	14 (5.3)	7 (2.7)	.285
** Intra-abdominal collections, *n* (%)**	3 (1.1)	5 (1.9)	5 (1.9)	.906
**Exam result**				
** Pathological exam, *n* (%)**	157 (59.5)	155 (58.7)	160 (60.6)	.914
**Appendix findings**				
** Visualizable appendix, *n* (%)** ^a^	29 (29.3)	21 (19.3)	26 (28.0)	.236
** Pathological appendix, *n* (%)** ^a^	1 (1.0)	1 (0.9)	0 (0.0)	.610
** Appendix thickness (mm), mean ± SD**	2.23 ± 1.32	3.39 ± 1.88	3.13 ± 0.92	.353

a Period 1: unsupervised examinations 31-63; Period 2: unsupervised examinations 64-96; Period 3: unsupervised examinations 97-129. Data are presented as mean ± standard deviation (SD) or number (percentage) as appropriate. Appendix findings are calculated based on patients where appendix visualization was attempted (Period 1: *n* = 99; Period 2: *n* = 109; Period 3: *n* = 93).

Basic pathological findings demonstrated consistent prevalence rates. Pathologically increased BWT (>3 mm) was observed in 57.6-61.0% of examinations, while increased vascularization was detected in 35.2-39.0% of cases. Advanced pathological findings occurred with substantially lower frequency but maintained consistent prevalence across periods. Fistulas were identified in 2.7-5.3% of examinations, intra-abdominal collections in 1.1-1.9%, free peritoneal fluid in 8.0-11.0%, and pathological dilation in 3.0-6.1% of cases.

### 3.2. Concordance between trainees and expert operators

Following completion of the initial theoretical training and the supervised performance of 30 IUS examinations with an expert operator, interobserver agreement between trainees and the expert was already substantial to excellent for most basic variables, with Cohen’s kappa coefficients exceeding 0.75 for seven of 12 individual parameters in their first 33 independent examinations. The results related to these findings are presented in Table 2.

**Table 2.** Agreement between trainees and expert sonographers in intestinal ultrasound parameters across training periods, after initial training (theory and 30 supervised exams) in trainees with prior abdominal ultrasound experience.

Parameter	Overall *κ* (95% CI)	Period 1 *κ* (95% CI)	Period 2 *κ* (95% CI)	Period 3 *κ* (95% CI)	Improvement (95% CI)	Raw *P*-value
**Basic parameters (Group)**	0.820 (0.776-0.863)	0.792 (0.743-0.842)	0.824 (0.774-0.875)	0.842 (0.795-0.890)	6.3% (2.1-10.5%)	.050 (FDR *P*-value .218)
**Pathologic segment identification**	0.890 (0.821-0.959)	0.875 (0.761-0.989)	0.851 (0.775-0.926)	0.946 (0.899-0.993)	8.1% (2.1-14.1%)	.078
**Bowel wall thickness**	0.854 (0.814-0.893)	0.830 (0.756-0.903)	0.877 (0.814-0.939)	0.854 (0.785-0.923)	2.9% (−3.7 to 9.5%)	.612
**Bowel wall stratification**	0.711 (0.628-0.793)	0.717 (0.577-0.858)	0.682 (0.531-0.832)	0.736 (0.599-0.872)	2.6% (−8.2 to 13.4%)	.883
**Vascularization (qualitative)**	0.865 (0.825-0.904)	0.856 (0.785-0.926)	0.863 (0.795-0.930)	0.875 (0.809-0.941)	2.2% (−4.8 to 9.2%)	.726
**Vascularization (Limberg score)**	0.799 (0.760-0.839)	0.745 (0.669-0.820)	0.820 (0.755-0.885)	0.833 (0.770-0.896)	11.9% (2.5-21.3%)	.076
**Mesenteric hypertrophy**	0.820 (0.772-0.868)	0.809 (0.721-0.897)	0.808 (0.725-0.890)	0.843 (0.763-0.923)	4.2% (−6.8 to 15.2%)	.635
**Enlarged lymph nodes**	0.798 (0.739-0.858)	0.713 (0.589-0.836)	0.870 (0.787-0.953)	0.808 (0.707-0.910)	13.5% (−2.4 to 29.4%)	.255
**Advanced parameters (Group)**	0.800 (0.746-0.855)	0.728 (0.649-0.806)	0.808 (0.710-0.906)	0.854 (0.804-0.905)	17.3% (6.8-27.8%)	.041 (FDR *P*-value .218)
**Free peritoneal fluid**	0.708 (0.609-0.807)	0.598 (0.386-0.810)	0.740 (0.589-0.890)	0.770 (0.614-0.927)	28.8% (1.2-56.4%)	.285
**Small bowel dilation**	0.784 (0.667-0.900)	0.674 (0.452-0.896)	0.824 (0.654-0.995)	0.921 (0.766-1.000)	36.6% (18.3-54.9%)	.017 (FDR *P*-value .218)
**Fistulas**	0.870 (0.767-0.974)	0.763 (0.496-1.000)	0.954 (0.865-1.000)	0.829 (0.593-1.000)	8.7% (−21.8 to 39.2%)	.627
**Intra-abdominal collections**	0.797 (0.599-0.995)	0.798 (0.403-1.000)	0.663 (0.197-1.000)	0.887 (0.665-1.000)	11.1% (−35.2 to 57.4%)	.989
**Overall pathologic findings**	0.843 (0.802-0.883)	0.805 (0.728-0.883)	0.860 (0.793-0.926)	0.864 (0.797-0.930)	7.3% (0.8-13.8%)	.240

Values represent Cohen’s kappa coefficients with 95% confidence intervals in parentheses. Raw *P*-values are from Cochran–Armitage trend tests; FDR *P*-values adjusted for multiple comparisons using the Benjamini–Hochberg method. Period 1: unsupervised examinations 31-63; Period 2: unsupervised examinations 64-96; Period 3: unsupervised examinations 97-129.

For basic parameters, overall agreement increased progressively from *κ* = 0.792 (95% CI: 0.743-0.842) in Period 1 to *κ* = 0.842 (95% CI: 0.795-0.890) in Period 3, representing a 6.3% relative improvement (95% CI: 2.1-10.5%) over the 66-examination training period.

Among individual basic parameters, identification of pathologic segments emerged as the most readily acquired skill, achieving excellent agreement by Period 3 (*κ* = 0.946, 95% CI: 0.899-0.993). BWT measurement demonstrated consistently high agreement across all periods (*κ* = 0.830-*κ* = 0.854).

Vascularization assessment showed distinct patterns between assessment methods. Qualitative vascularization assessment maintained consistently high agreement throughout training (from *κ* = 0.856 to *κ* = 0.875), while the Limberg score demonstrated significant improvement potential, progressing from *κ* = 0.745 (95% CI: 0.669-0.820) to *κ* = 0.833 (95% CI: 0.770-0.896). BWS emerged as the most challenging basic parameter, with agreement levels showing minimal improvement throughout the study period (*κ* = 0.717 to *κ* = 0.736).

Mesenteric feature assessment revealed divergent patterns: hypertrophy evaluation remained stable with good agreement, while enlarged mesenteric lymph node detection showed variable performance, with notable improvement from Period 1 (*κ* = 0.713, 95% CI: 0.589-0.836) to Period 3 (*κ* = 0.808, 95% CI: 0.707-0.910).

More pronounced improvements were observed for advanced variables, which increased from *κ* = 0.728 (95% CI: 0.649-0.806) in Period 1 to *κ* = 0.854 (95% CI: 0.804-0.905) in Period 3. Most improvements for advanced variables occurred during the latter half of the training period (examinations 64–129).

Assessment of the overall pathological findings, representing the most clinically relevant outcome for integrated decision-making, improved from *κ* = 0.805 (95% CI: 0.728-0.883) to *κ* = 0.864 (95% CI: 0.797-0.930) over the study period (detailed in Table 2).

**Figure 1.**
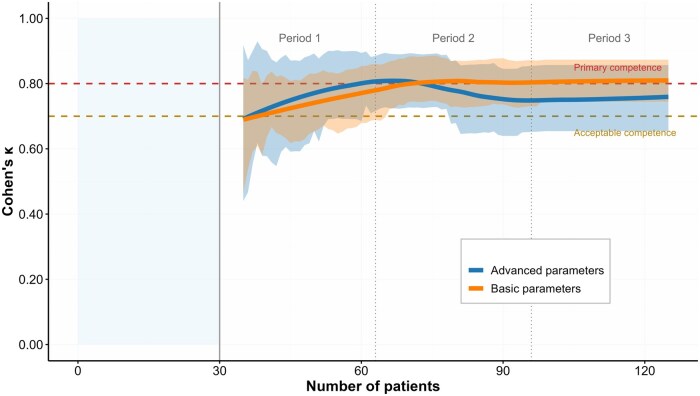
Learning curves for basic and advanced intestinal ultrasound parameters after the initial training phase, which included a theoretical module and 30 supervised examinations performed with an expert in bowel ultrasound, in trainees already experienced in abdominal ultrasound. *Note*: the initial “Supervised Training” period (examinations 1-30) represents the structured hands-on training phase where trainees performed examinations under direct expert guidance. The subsequent three periods (Period 1: examinations 31-63; Period 2: examinations 64-96; Period 3: examinations 97-129) represent independent assessments with progressive skill development. The orange line represents basic parameters (bowel wall thickness, stratification, vascularization assessment, Limberg score, mesenteric features), while the blue line represents advanced parameters (fistulas, collections, dilation, free fluid). Horizontal dashed lines indicate acceptable competence (*κ* = 0.7, orange) and primary competence (*κ* = 0.8, red) thresholds. Shaded areas represent 95% confidence intervals.

**Figure 2.**
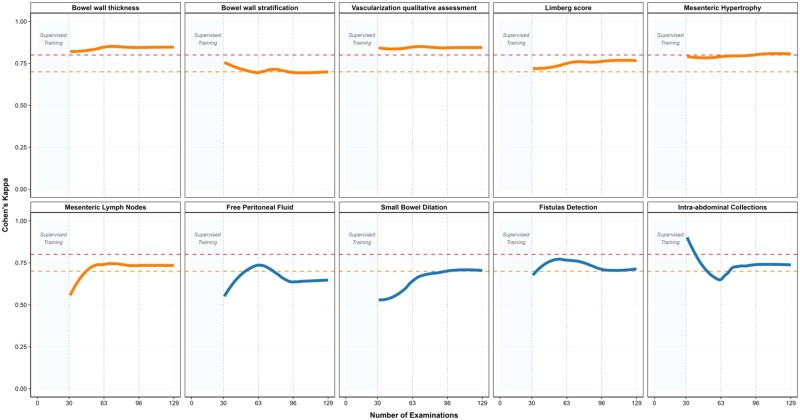
Learning curves for intestinal ultrasound parameter types after the initial training phase, which included a theoretical module and 30 supervised examinations performed with an expert in bowel ultrasound, in trainees already experienced in abdominal ultrasound. *Note:* the initial “Supervised Training” period (examinations 1-30) represents the structured hands-on training phase where trainees performed examinations under direct expert guidance. The subsequent three periods (Period 1: examinations 31-63; Period 2: examinations 64-96; Period 3: examinations 97-129) represent independent assessments with progressive skill development. The blue line represents basic parameters (bowel wall thickness, stratification, vascularization assessment, Limberg score, mesenteric features), while the orange line represents advanced parameters (fistulas, collections, dilation, free fluid). Horizontal dashed lines indicate acceptable competence (*κ* = 0.7, orange) and primary competence (*κ* = 0.8, red) thresholds. Shaded areas represent 95% confidence intervals. Note that the apparent visual narrowing in some curves reflects the confidence interval convergence as trainees’ performance stabilized, not a decline in actual performance. The kappa values for advanced parameters improved from 0.728 to 0.854 across the training period.

Small bowel dilation detection exhibited the most impressive learning trajectory, improving from *κ* = 0.674 (95% CI: 0.452-0.896) in Period 1 to *κ* = 0.921 (95% CI: 0.766-1.000) in Period 3. Similarly, free peritoneal fluid detection showed substantial and consistent improvement from *κ* = 0.598 (95% CI: 0.386-0.810) to *κ* = 0.770 (95% CI: 0.614-0.927). The differences between advanced and basic variables are illustrated in Figures 1 and 2.

Prevalence-adjusted analysis revealed minimal bias for most IUS variables, with only two of 12 parameters (16.7%) demonstrating clinically meaningful prevalence effects requiring interpretation adjustment. Fistula detection and intra-abdominal fluid collections required adjustments (*κ* = 0.663 vs PABAK = 0.833, difference = 0.170 vs *κ* = 0.826 vs PABAK = 0.939, ­difference = 0.113, respectively).

### 3.3. Learning curve characteristics

Our analysis revealed fundamentally distinct learning patterns between basic and advanced ultrasound variables. Basic parameters exhibited high baseline performance (mean *κ* = 0.792) with modest incremental gains (0.025 kappa units per period). Conversely, advanced parameters demonstrated lower initial competency (mean *κ* = 0.728) but substantially steeper learning trajectories (0.063 kappa units per period), indicating these complex diagnostic skills require dedicated practice beyond foundational ultrasound training. No individual parameter improvements achieved statistical significance (all FDR-adjusted *P* > .20).

Hierarchical modeling revealed substantial heterogeneity in learning patterns, with between-trainee variance explaining 0-48% of the total variation depending on parameter complexity. Subjective evaluations including mesenteric hypertrophy (ICC = 0.479) and intra-abdominal collections (ICC = 0.416) exhibited high inter-operator variability, while small bowel dilation showed minimal clustering (ICC = 0.038), indicating consistent improvement across operators. Mixed-effects analysis confirmed that while small bowel dilation showed the largest positive coefficient for Period 3 (β_3_ = +2.092), no individual variables achieved statistical significance after FDR correction for multiple comparisons (all FDR *P* > .38).

**Figure 3.**
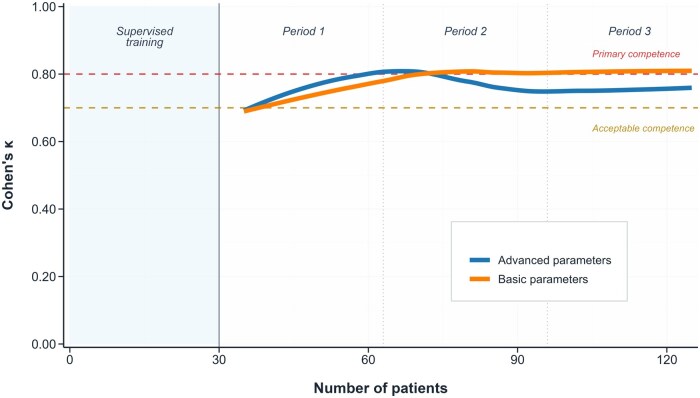
Learning rates for parameters with low agreement after initial training (theory and 30 supervised exams) in trainees with prior abdominal ultrasound experience.

We employed dual analytical approaches to characterize learning dynamics. Linear modeling quantified absolute skill acquisition rates, while logarithmic regression identified variables following classical learning curves with rapid initial gains followed by performance plateaus (Table 3; Figure 3).

**Table 3.** Learning rate analysis and difficulty categorization for IUS parameters after initial training (theory and 30 supervised exams) in trainees with prior abdominal ultrasound experience.

Parameter	Period 1 *κ* (95% CI)	Period 3 *κ* (95% CI)	Absolute improvement (95% CI)	**Linear learning rate** ^a^**(95% CI)**	Percentage improvement (95% CI)	**Logarithmic rate** ^b^**(95% CI)**	Difficulty category	*P*-value
**Basic parameters**								
** Pathologic segment identification**	0.875 (0.761-0.989)	0.946 (0.899-0.993)	0.071 (0.018-0.124)	0.036 (0.009-0.062)	8.1% (2.1-14.1%)	0.054 (0.012-0.096)	High-proficiency	.585
** Bowel wall thickness**	0.830 (0.756-0.903)	0.854 (0.785-0.923)	0.024 (−0.031 to 0.079)	0.012 (−0.016 to 0.040)	2.9% (−3.7 to 9.5%)	0.027 (−0.018 to 0.072)	High-proficiency	.559
** Bowel wall stratification**	0.717 (0.577-0.858)	0.736 (0.599-0.872)	0.018 (−0.059 to 0.095)	0.009 (−0.030 to 0.048)	2.6% (−8.2 to 13.4%)	0.009 (−0.045 to 0.063)	Intermediate-complexity	.878
** Vascularization (qualitative)**	0.856 (0.785-0.926)	0.875 (0.809-0.941)	0.019 (−0.041 to 0.079)	0.010 (−0.021 to 0.040)	2.2% (−4.8 to 9.2%)	0.017 (−0.031 to 0.065)	High-proficiency	.201
** Limberg score**	0.745 (0.669-0.820)	0.833 (0.770-0.896)	0.088 (0.019-0.157)	0.044 (0.010-0.078)	11.9% (2.5-21.3%)	0.083 (0.018-0.148)	Intermediate-complexity	.151
** Mesenteric hypertrophy**	0.809 (0.721-0.897)	0.843 (0.763-0.923)	0.034 (−0.055 to 0.123)	0.017 (−0.028 to 0.062)	4.2% (−6.8 to 15.2%)	0.027 (−0.042 to 0.096)	High-proficiency	.455
** Enlarged lymph nodes**	0.713 (0.589-0.836)	0.808 (0.707-0.910)	0.096 (−0.017 to 0.209)	0.048 (−0.009 to 0.105)	13.5% (−2.4 to 29.4%)	0.102 (−0.012 to 0.216)	Intermediate-complexity	.493
**Advanced parameters**								
** Free peritoneal fluid**	0.598 (0.386-0.810)	0.770 (0.614-0.927)	0.172 (0.007-0.337)	0.086 (0.004-0.169)	28.8% (1.2-56.4%)	0.162 (0.005-0.319)	High-challenge	.131
** Small bowel dilation**	0.674 (0.452-0.896)	0.921 (0.766-1.000)	0.247 (0.108-0.386)	0.123 (0.054-0.193)	36.6% (18.3-54.9%)	0.224 (0.096-0.352)	High-challenge	.017 (FDR-adjusted *P*-value .204)
** Fistulas**	0.763 (0.496-1.000)	0.829 (0.593-1.000)	0.066 (−0.165 to 0.297)	0.033 (−0.083 to 0.149)	8.7% (−21.8 to 39.2%)	0.084 (−0.124 to 0.292)	Intermediate-complexity	.684
** Intra-abdominal collections**	0.798 (0.403-1.000)	0.887 (0.665-1.000)	0.089 (−0.281 to 0.459)	0.044 (−0.141 to 0.230)	11.1% (−35.2 to 57.4%)	0.051 (−0.198 to 0.300)	Initially easy, maintained	.838
**Overall pathologic findings**	0.805 (0.728-0.883)	0.864 (0.797-0.930)	0.058 (−0.007 to 0.123)	0.029 (−0.004 to 0.062)	7.2% (−0.9 to 15.3%)	0.056 (−0.008 to 0.120)	High-proficiency	.200

Values represent Cohen’s kappa coefficients with 95% confidence intervals in parentheses. Raw *P*-values are from Cochran–Armitage trend tests; FDR *P*-values adjusted for multiple comparisons using the Benjamini–Hochberg method.

a Linear learning rate = average improvement in kappa coefficient per training period (calculated as [Period 3 *κ*−Period 1 *κ*]/2).

b Logarithmic learning rate = coefficient from logarithmic regression model (*κ* ∼ log(period)), representing the steepness of the learning curve. Period 1: unsupervised examinations 31-63; Period 3: unsupervised examinations 97-129.

High-proficiency variables (*κ* > 0.8 baseline) including variables such as pathologic segment identification and BWT measurement demonstrated ceiling effects, with learning rates of 0.009-0.036 kappa units per period.

Intermediate-complexity variables (*κ* = 0.7–0.8 baseline) included the Limberg score and lymph node evaluation.

High-challenge variables (*κ* < 0.7 baseline) include free peritoneal fluid detection, which achieved 28.8% relative improvement with the highest linear learning rate (0.086 kappa units per period).

### 3.4. Competency achievement analysis

Competency development was assessed using two distinct methodologies: kappa-based evaluation measuring the quality of diagnostic agreement with expert assessment, and CUSUM analysis evaluating binary detection capability. These approaches revealed variable-specific competency patterns that varied significantly based on the prevalence of pathological findings and the nature of the diagnostic task.

Primary competence achievement varied substantially across ultrasound variables. Common pathological findings demonstrated the highest success rates: identification of pathologic segments, BWT measurement, and qualitative vascularization assessment each achieved primary competence in 5/8 trainees (62.5%). These data are presented in Table 4.

**Table 4.** Cohen’s kappa threshold competency achievement analysis for intestinal ultrasound parameters after initial training (theory and 30 supervised exams) in trainees with prior abdominal ultrasound experience.

Parameter	Primary competence achievement (95% CI)	Primary: mean ± SD	Primary: range	Acceptable competence achievement (95% CI)	Acceptable: mean ± SD	Acceptable: range
**Identification of ­pathologic segment**	5/8 (62.5%, 24.5-91.5%)	54.2 ± 23.7	35-82	8/8 (100.0%, 63.1-100.0%)	59.3 ± 31.3	35-113
**Bowel wall thickness**	5/8 (62.5%, 24.5-91.5%)	64.4 ± 39.1	35-126	6/8 (75.0%, 34.9-96.8%)	50.8 ± 33.3	35-118
**Vascularization (qualitative)**	5/8 (62.5%, 24.5-91.5%)	57.6 ± 36.6	35-121	6/8 (75.0%, 34.9-96.8%)	42.5 ± 9.8	35-57
**Overall pathologic findings**	3/8 (37.5%, 8.5-75.5%)	53.8 ± 37.5	35-110	4/8 (50.0%, 15.7-84.3%)	39.4 ± 7.8	35-53
**Vascularization (Limberg score)**	4/8 (50.0%, 15.7-84.3%)	48.0 ± 22.8	35-82	4/8 (50.0%, 15.7-84.3%)	42.2 ± 11.4	35-59
**Fistulas**	4/8 (50.0%, 15.7-84.3%)	56.2 ± 24.6	35-80	4/8 (50.0%, 15.7-84.3%)	39.5 ± 7.3	35-52
**Mesenteric hypertrophy**	3/8 (37.5%, 8.5-75.5%)	55.5 ± 16.5	38-71	3/8 (37.5%, 8.5-75.5%)	51.5 ± 13.0	38-68
**Bowel wall stratification**	3/8 (37.5%, 8.5-75.5%)	82.3 ± 26.7	52-102	4/8 (50.0%, 15.7-84.3%)	66.8 ± 24.9	40-93
**Enlarged mesenteric lymph nodes**	4/8 (50.0%, 15.7-84.3%)	45.0 ± 7.2	37-51	5/8 (62.5%, 24.5-91.5%)	45.0 ± 7.2	37-51
**Free peritoneal fluid**	4/8 (50.0%, 15.7-84.3%)	74.3 ± 42.9	35-120	6/8 (75.0%, 34.9-96.8%)	69.5 ± 27.1	35-101
**Small bowel dilation**	3/8 (37.5%, 8.5-75.5%)	69.3 ± 30.0	36-94	4/8 (50.0%, 15.7-84.3%)	54.7 ± 21.4	36-78
**Intra-abdominal fluid collections**	3/8 (37.5%, 8.5-75.5%)	50.0 ± 0.0	50	3/8 (37.5%, 8.5-75.5%)	50.0 ± 0.0	50

Competency achievement analysis used predefined kappa thresholds. Primary competence was defined as *κ* ≥ 0.80 (excellent agreement), acceptable competence as *κ* ≥ 0.70 (substantial agreement). Competency required reaching threshold and maintaining performance throughout all remaining examinations. Cases to competency are reported as examination numbers within the unsupervised training period (examinations 31-129).

Intermediate-complexity variables showed moderate achievement rates, with overall pathologic findings assessment, Limberg score evaluation, fistula detection, and mesenteric hypertrophy assessment each reaching primary competence in 3/8 trainees (50.0%).

Challenging variables demonstrated lower success rates: BWS, enlarged mesenteric lymph node detection, free peritoneal fluid assessment, and small bowel dilation evaluation each achieved primary competence in only 3/8 trainees (37.5%). Intra-abdominal fluid collections emerged as the most difficult variable, with sustained primary competence achieved by only 3/8 trainees (12.5%).

Among trainees who successfully achieved primary competence, examination requirements within the independent training period varied considerably. Free peritoneal fluid detection demanded the most practice (74.3 ± 42.9 examinations), followed by small bowel dilation assessment (69.3 ± 30.0 examinations), and overall pathologic findings evaluation (53.8 ± 37.5 examinations). Conversely, enlarged mesenteric lymph nodes required fewer examination attempts (45.0 ± 7.2 examinations each) despite their low overall achievement rates.

Acceptable competence (*κ* ≥ 0.7) demonstrated, as expected, substantially higher success rates across all variables. Pathologic segment identification achieved universal competence while BWT, qualitative vascularization, and overall pathologic findings each reached 75% success (6/8 trainees). Most remaining variables achieved 50-62.5% acceptable competence rates, indicating that substantial agreement with expert operators represents an attainable training goal for the majority of ultrasound assessments.

## 4. Discussion

This multicenter study provides the first comprehensive quantitative framework for IUS competency acquisition in IBD, encompassing 792 assessments across 12 clinically validated variables. Competence in IUS is a multidimensional construct encompassing theoretical knowledge, image acquisition skills, and image interpretation, as defined by the Delphi consensus on IUS training standards.^13^ Our study focused primarily on the interpretative dimension, using diagnostic agreement with expert operators as a measurable proxy for clinical competence. Rather than fundamentally challenging established paradigms, our findings contribute to redefining current IUS training models by demonstrating that structured education can achieve expert-level proficiency within 75-112 examinations. Following theoretical preparation and 30 supervised examinations, trainees progressed from *κ* = 0.792 to 0.842 for basic and from *κ* = 0.728 to 0.854 for advanced parameters, achieving primary competence (*κ* ≥ 0.8) in up to 62.5% of cases.

Our results align with recent IUS learning curve research while providing important parameter-specific refinements. Our analysis revealed distinct learning phenotypes transcending conventional categorizations. Small bowel dilation demonstrated the most dramatic improvement trajectory (from *κ* = 0.674 to 0.921, 36.6% enhancement) despite advanced classification, while BWS showed minimal progression (from *κ* = 0.717 to 0.736, 2.6% improvement) despite basic categorization. The absence of significant learning curves in most basic variables probably reflects ceiling effects due to effective theoretical training and supervised practice phases. In fact, recent data suggest that even within “basic” variables, substantial complexity exists. Our findings complement and expand upon those of Madsen et al.,^12^ who evaluated learning curves for BWT and echostratification, while our analysis encompasses both basic and advanced pathological findings, including rare complications such as fistulas and abscesses. Madsen et al. similarly demonstrated that BWT accuracy improved from 75% to 85% after 80 cases when working with expert-acquired images while the identification of BWS remained challenging to master.^17^ This persistent challenge with stratification assessment was also identified by Bove et al., suggesting this parameter requires targeted educational approaches beyond simple case exposure.^18^ A contemporary multicenter reliability study revealed only fair-to-moderate agreement for morphological features including stratification among experts,^11^ a finding that directly correlates with our learning trajectories. Conversely, in our study high inter-operator variability was characteristic of mesenteric hypertrophy (ICC = 0.479) and collections (ICC = 0.416). The correlation between expert reliability limitations and trainee learning difficulties often reflects inherent interpretive challenges persisting across experience levels, fundamentally reframing competency expectations.

Most critically, advanced variables showed substantially lower baseline agreement, with free peritoneal fluid detection at *κ* = 0.598 and small bowel dilation at *κ* = 0.674. These findings also align with our previous study,^15^ which examined gastroenterologists with varying ultrasound experience and established that 84 examinations were required for accurate BWT measurement.

Regarding learning curve trajectories, the logarithmic learning rate analysis revealed that variables with rates >0.08 (small bowel dilation, enlarged lymph nodes, Limberg score) followed steep initial learning curves that gradually plateaued, consistent with typical skill acquisition patterns. The most striking individual finding improvement was observed in small bowel dilation detection, which advanced from *κ* = 0.674 to *κ* = 0.921 (36.6% relative improvement), demonstrating that complex diagnostic skills can be acquired with sufficient practice. Free peritoneal fluid detection showed the steepest learning trajectory among advanced assessment, improving by 28.7% from *κ* = 0.598 to *κ* = 0.770. The less steep improvement in the Limberg score might follow similar trends that have been observed in imaging studies, where composite scoring systems require more deliberate practice for mastery.^26^^,^^27^ The lack of statistical significance for improvement in all variables, despite numerical gains, may stem from small sample size (*n* = 8), high initial agreement (*κ* > 0.8) leading to ceiling effects, and the low prevalence of certain complications (eg, fistulas, abscesses).

CUSUM analysis demonstrated uniformly high apparent competency achievement rates, with 75% of trainees (6/8) reaching binary detection competency within 48.5-61.6 examinations across evaluated variables (Table SII). However, CUSUM findings require careful methodological interpretation due to inherent limitations of this method when applied to rare pathological conditions, where the binary decision framework of CUSUM analysis creates a fundamental bias toward apparent “ease” for low-prevalence findings. Consequently, we restricted CUSUM analysis to variables with prevalence ≥10%, ensuring meaningful interpretation of binary detection capabilities while avoiding the statistical artifacts associated with rare findings.

The convergence of competency rates (75%) across diverse variables in CUSUM analysis, contrasting with the substantial variation observed in kappa-based assessments (12.5%-62.5% primary competence), underscores the fundamental difference between binary detection capability and nuanced diagnostic agreement quality. This distinction has important implications for competency assessment methodology: CUSUM effectively evaluates basic detection skills essential for screening applications, while kappa-based analysis captures the sophisticated interpretive agreement required for clinical decision-making.

The parameter-specific learning curves identified in our study have significant implications for educational program design. Our data support a competency-based rather than time-based approach to IUS training. A staggered learning approach, introducing complex semi-quantitative assessments after trainees establish foundational skills, could enhance learning efficiency and skill retention. Standardized vascularity assessments, such as the Limberg score, lymph node detection, as well as the loss of bowel stratification, may benefit from focused educational interventions, such as targeted image review sessions emphasizing vascularity gradations. The differential complexity and learning curves identified in our study have important implications for the global implementation of IUS in IBD management, particularly in regions where adoption has been limited, such as North America and the Asia-Pacific region.^28^^,^^29^ By providing realistic expectations for training requirements and competency development, our findings may help institutions develop feasible implementation strategies that account for the resources required to achieve diagnostic reliability.

Our investigation has several distinctive methodological strengths that enhance the validity and clinical relevance of our findings. This study represents the first comprehensive evaluation of IUS learning curves based on complete dynamic ultrasound procedures rather than static image interpretation. Our assessment encompassed both critical components of ultrasound competency: (1) image acquisition and (2) image interpretation. Although image acquisition was included as part of the practical training process, our evaluation did not formally quantify acquisition quality or probe-handling skills, which represent distinct components of IUS competence. This methodological distinction has relevant implications for medical education and for the future integration of artificial intelligence in ultrasound training.

Second, this study assessed the learning curve for all intestinal and extra-intestinal variables that are commonly evaluated during an IUS.

While our study provides robust data on IUS learning curves, some limitations should be noted. First, our cohort had prior abdominal ultrasound experience (>200 examinations), limiting generalizability to different operator backgrounds. Second, the sample size of eight trainees may not capture full learning variability. Low prevalence of advanced findings limited exposure to rare complications. While we successfully characterized learning curves for different parameters, our results provide a more nuanced answer to establishing the minimum number of examinations. Rather than identifying a single minimum number of examinations, our data reveal parameter-specific requirements ranging from 45 to 112 examinations, supporting a competency-based approach. Third, the study was conducted in Italian tertiary centers with established IUS expertise, limiting generalizability. We assessed interobserver agreement rather than diagnostic accuracy against gold standards. While interobserver agreement with expert operators provides an objective measure of interpretative accuracy, it may not fully capture the multidimensional nature of competence. Inter-expert variability in IUS interpretation remains a known challenge and should be ­considered when using kappa values as a surrogate for skill acquisition.

Future research should evaluate learning trajectories in diverse training populations and assess long-term skill retention. Investigating optimal refresher training intervals and competency maintenance requirements would inform ongoing professional development standards. Furthermore, correlating trainee assessments with endoscopic or cross-sectional imaging findings would strengthen the evidence base for diagnostic accuracy benchmarks. Despite the limited number of trainees, the multicenter design and standardized supervision across eight expert centers ensured consistency and enhanced the generalizability of our observations. A fundamental consideration in our methodology is the inherent variability in expert assessment itself. De Voogd et al. demonstrated only fair-to-moderate inter-rater reliability among experts for morphological features including stratification. This raises important questions about using expert agreement as the sole benchmark for competency. The persistent challenges our trainees experienced with BWS assessment (achieving only *κ* = 0.736) may reflect inherent interpretive ambiguity rather than inadequate training. Future competency frameworks might benefit from incorporating acceptable ranges of variation rather than absolute agreement thresholds.^11^ Moreover, while our study evaluated concordance in findings between trainees and experts, suggesting adequate image acquisition skills, we did not formally assess specific technical competencies including transducer handling, ergonomics, or machine optimization. These components of the competency framework described by the Delphi consensus represent important areas for future investigation.

## 5. Conclusion

This study establishes the first comprehensive, variable-specific learning thresholds for IUS competency in IBD management. Our findings demonstrate that structured training enables basic IUS proficiency within 69-112 examinations, while advanced skills require extended practice with individualized support.

These findings represent an important step toward evidence-based IUS training standards, providing quantitative reference points that can inform—but not yet replace—competency-based certification frameworks.

## Data Availability

The data underlying this article will be shared on reasonable request to the corresponding author.
